# Rapid and selective recovery of palladium from platinum group metals and base metals using a thioamide-modified calix[4]arene extractant in environmentally friendly hydrocarbon fluids

**DOI:** 10.1038/s41598-018-35026-x

**Published:** 2018-11-15

**Authors:** Manabu Yamada, Muniyappan Rajiv Gandhi, Atsushi Shibayama

**Affiliations:** 1 Research Center of Advanced Materials for Breakthrough Technology, Graduate School of Engineering Science, Akita University, Akita, 010-8502 Japan; 2 Graduate School of International Resource Sciences, Akita University, Akita, 010-8502 Japan; 3 Present Address: Quality Control Department, Panipat Refinery & Petrochemical Complex, Indian Oil Corporation Limited, Haryana, 132140 India

## Abstract

A novel macrocyclic calix[4]arene extractant having a long alkyl chain thioamide, 25,26,27,28-tetrakis(*N*-*n*-octylthiocarbamoyl)methoxy-5,11,17,23-tetra-*tert*-butylcalix[4]arene (**1**), was synthesized from 25,26,27,28-tetrakis(*N*-*n*-octylcarbamoyl)methoxy-5,11,17,23-tetra-*tert*-butylcalix[4]arene (**2**) using Lawesson’s reagent. Extractant **1** was characterized using ^1^H NMR, ^13^C NMR, FT-IR spectroscopy, and elemental analysis. The Pd(II) extraction abilities of **1** and **2** were studied in high-boiling-point and environmentally friendly hydrocarbon diluents. Pd(II) extraction experiments were conducted using single-metal Pd(II) solutions, simulated mixed palladium group metal (PGM) solutions, and acid-leached automotive catalyst residue solutions. Different experimental conditions, including the shaking time, HCl/HNO_3_ concentration, Pd(II) concentration, extractant concentration, and the organic/aqueous phase ratio, were studied systematically. Extractant **1** showed very selective (> 99.9%) Pd(II) extraction from the mixed PGM/base metal solutions and the acid-leached automotive catalyst residue solution. Conversely, the Pd(II) extraction ability of extractant **2** was found to be negligible. Extractant **1** showed very fast extraction kinetics and a high extraction capacity as compared to those of the commercial extractant di-*n*-octyl sulfide. Effective stripping of Pd(II) from **1** was performed using HCI, HNO_3_, NH_3_, and HCl-thiourea solutions. Furthermore, **1** was successfully recycled over five extraction/stripping cycles. The Pd(II) extraction mechanism of **1** was studied using FT-IR spectroscopy. Extractant **1** exhibited very selective Pd(II) extraction and high acid stability, demonstrating its industrial applicability for the extraction of Pd(II) from leached automotive catalyst liquors containing PGMs and base metals.

## Introduction

The concentration of platinum group metals (PGMs) in Earth’s crust is approximately 0.001%, and it is localized in particular countries such as South Africa and Russia^1,2^. A stable supply of PGMs is crucial because of the difficulties associated with their extraction and their high cost. Furthermore, their global market prices fluctuate with demand^3^. The primary production of PGMs generates large amounts of mining waste, consumes large quantities of energy and water, and produces potentially hazardous exhaust flue gases such as CO_2_ and SO_2_^4^. Individual PGM separation and refining consume significant quantities of water and acidic substances, which pollute both water and land^4^. Currently, 40% of the Pt, 58% of the Pd, and 83% of the Rh produced globally are used in the automobile industry for catalytic converters^5^. The calculated energy requirements for refining Rh (683,000 MJ/kg), Pt (243,000 MJ/kg), and Pd (72,700 MJ/kg) are much larger than that for steel, which requires <35 MJ/kg^4^. Thus, PGM mining and purification present enormous environmental burdens. Recycling of PGMs from waste exhaust catalysts, industrial catalysts, and e-waste is economically important, reduces the environmental burden of metal mining, and can limit environmental pollution^6^. Spent catalytic converters contain PGMs at levels up to 2‒10 g/kg, which is significantly higher than those in primary ores (~0.01 g/kg)^6,7^. At present, the separation and recovery of high-value PGMs from end-of-life products (secondary resources) have become a worldwide necessity. Among the PGMs, Pd(II) is widely used in automotive catalysts for exhaust-gas-emission control in gasoline engines^3^. The recycling of PGMs from automotive catalysts is very important for ensuring their supply chain and maintaining their circular economies. In general, hydrometallurgical processes, spent catalytic converters are cut open and then their inner core materials are crushed into powders^8^. Finally, these powders are leached with HCl and HNO_3_ solutions containing oxidizing agents^8^. PGMs have been separated from acid-leached catalyst residue solutions using a range of commercial extractants^9–11^. For example, di-*n*-alkyl sulfide (DAS), 2-hydroxy-5-nonylacetophenone oxime (LIX 84A), 5,8-diethyl-7-hydroxydodecan-6-oxime (LIX 63), di-2-ethylhexylphosphoric acid (D2EHPA), PC88A, Cyanex 272/301, tri-*n*-octyl phosphine oxide (TOPO), β-diketones, and tri-*n*-butyl phosphate (TBP) have been used extensively in solvent extraction processes for Pd(II)^1,2,12,13^. These extractants are effective for the separation of PGMs. However, they degrade over time, adversely affecting their extraction rates and metal-ion selectivities^14^. Concomitantly, these extractants are oxidized upon extended contact with highly acidic aqueous phases, rendering them ineffective for metal separation^10,11^. For example, LIX-type extractants are susceptible to hydrolysis under acidic conditions, which impairs the selectivity of the corresponding systems^1,2^. D2EHPA and Cyanex 272 undergo aqueous solubility/degradation in acidic media during metal extraction, and their degradation is mainly dependent on the acid concentration and pH of the aqueous phase^15^. The kinetics of Pd(II) extraction using LIX 84A and DAS are extremely slow^1,2,11^. DAS is oxidized to di-*n*-alkyl sulfoxide (DASO) during extraction upon contact with oxidizing agents in the acidic aqueous phase, decreasing its Pd(II) extraction selectivity^11^. Therefore, a new generation of extractants with higher extraction rates, high metal selectivities, and superior durabilities is required.

Calix[*n*]arenes (where *n* = 4–20) are bowl-shaped macrocyclic compounds that have been widely used in catalysis, molecular recognition, metal ion separation, and sensors^16^. Calix[*n*]arenes have varying metal-recognition abilities that can be tuned by introducing different functional groups to the upper and lower rims. Calix[4]arenes are readily synthesized by a facile one-pot procedure involving the condensation of HCHO with phenol^16,17^. Calix[4]arenes can be tailored to selectively bind specific PGMs using chemical modifications^18,19^. Thus, functionalized macrocyclic calix[4]arenes (basket-like molecules) can simplify the enrichment of PGMs and significantly decrease the use of excess extractants^20^. Our previous results showed that macrocyclic calixarene or thiacalixarene-based extractants and other new thiocarbamoyl-based extractants are more durable in acid media than commercial extractants and extract approximately 3–5-fold more metal ions than commercial extractants from secondary resource leach liquors^19,21–28^. Generally, calixarenes and their derivatives are highly soluble only in aromatic/chlorinated diluents such as chloroform, dichloromethane, dichloroethane, and toluene^29^. To date, experiments on the PGM extraction capabilities of calixarene derivatives have only been conducted in chlorinated diluents^19,20,29^. However, the use of chlorinated diluents has several disadvantages, including their low boiling points and issues related to environmental and health concerns during industrial operations. In the present study, our focus was to design and synthesize a novel long-alkyl-chain-thioamide-functionalized multifunctional calix[4]arene extractant that dissolves in high-boiling-point hydrocarbon diluents (kerosene, *n*-dodecane, ShellSol D70, ISOPAR M, *n*-octanol, Escaid^TM^ 110 fluid, Escaid^TM^ 110, and Exxal^TM^ 10) for the industrial separation of Pd(II) from automotive catalysts. Accordingly, the novel macrocyclic calix[4]arene extractant 25,26,27,28-tetrakis(*N*-*n*-octylthiocarbamoyl)methoxy-5,11,17,23-tetra-*tert*-butylcalix[4]arene (**1**) was synthesized from 25,26,27,28-tetrakis(*N*-*n*-octylcarbamoyl)methoxy-5,11,17,23-tetra-*tert*-butylcalix[4]arene (**2**) using Lawesson’s reagent. The synthesized extractant displayed high solubility in kerosene, Exxal^TM^ 10 (a branched alcohol diluent that is readily biodegradable), and also other liquid hydrocarbons. The Pd(II) extraction abilities of the synthesized extractants from single-metal Pd(II) solutions, mixed PGM/base metal solutions, and leach liquors of automotive catalyst residues in Cl^−^ media were evaluated extensively. Various experimental conditions, including shaking time, HCl/HNO_3_ concentration, diluent, extractant concentration, and metal ion concentrations were investigated. Extractant **1** showed rapid and selective extraction of Pd(II) (*E*% > 99%) from the single-metal solutions, simulated mixed PGM/base metal solutions, and leach liquors from automotive catalysts containing Rh, Pd, Pt, Zr, Ce, Ba, Al, La, and Y in Cl^−^ media. Furthermore, the stripping of Pd(II) from the resultant inclusion complexes and the reusability of extractant **1** were studied. The synthesized extractant **1** was found to be robust in acidic media, and it provided very fast and selective extraction of Pd(II) from highly acidic media and in the presence of base/other metals as compared to that achieved with the commercial extractant di-*n*-octyl sulfide (DOS). The current work is the first example of Pd(II) separation using the calixarene-based extractant **1** in hydrocarbon diluents. Furthermore, our results indicate that it may be applicable to current industrial refining processes.

## Results and Discussion

### Salient features of the extractants and the effect of diluent on the extraction of Pd(II)

In the current study, liquid-liquid extraction of metal ions using calixarene-based extractants was carried out in chlorinated or aromatic diluents (e.g., chloroform or toluene), which have low boiling and flash points. Due to the environmental and biological toxicities of chlorinated and aromatic diluents, they are not recommended for rare metal refining processes. In the present study, we fitted the calix[4]arene-based extractants **1** and **2** with *n*-octyl chains to increase their solubilities in high-boiling- and high-flash-point hydrocarbon-based diluents. The *n*-octyl groups also promote rapid organic/aqueous (O/A) phase separation during metal ion extraction.

The effect of diluent on the extraction of Pd(II) ions by **1** and **2** was assessed using seven aliphatic diluents and four aromatic/chlorinated diluents. Table 1 shows the effect of diluent on the extraction of Pd(II) with **1** and **2**. Extractant **1** shows emulsion formation during Pd(II) extraction (*E*% = 97‒98%) with the aliphatic diluents kerosene, ISOPAR M, ShellSol D70, *n*-dodecane, and Escaid^TM^ 110 (i.e., isodecyl alcohol, which exhibits no significant physical or chemical hazards)^30^. In order to prevent emulsion formation during extraction, *n*-octanol was mixed with the diluents above at 20 vol%. Extractant **1** shows good phase separation upon addition of *n*-octanol to kerosene, ISOPAR M, ShellSol D70, *n*-dodecane, and Escaid^TM^ 110 and exhibits Pd(II) *E*% < 99.7%. Among the studied diluents, kerosene containing 20% *n*-octanol shows very high Pd(II) extraction ability (*E*% = 99.81%) by **1**. The diluent *n*-octanol acts as a phase modifier and prevents emulsion formation during Pd(II) extraction. Figure S1 shows photographs of experimental Pd(II) extraction set-ups using **1** in kerosene alone and kerosene containing 20% *n*-octanol and **2** in kerosene alone. When **1** is diluted in the alcohol-based diluents Exxal^TM^ 10 and *n*-octanol, very clear phase separation is observed and the Pd(II) extraction is found to be < 99.6%. Similarly, the Pd(II) extraction ability of **1** in the aromatic and chlorinated diluents *p*-xylene, *o*-dichlorobenzene, toluene, and CHCl_3_ were assessed. Extractant **1** shows *E*% values of 96.7‒99.8% in the aromatic and chlorinated diluents with very clear phase separation, whereas extractant **2** shows very low Pd(II) extraction (*E*% < 2.3) in all diluents studied. We also performed control experiments using the diluents without the extractants.

**Table 1:** Effect of diluents on the extraction of Pd(II) by **1** and **2**.

Aliphatic Diluents	Boiling point (°C)	Flash point (°C)	Pd(II) Extraction (%)			
			1	Remarks	2	Remarks
Kerosene	150–300	38–66	98.26	Emulsion	1.38	CPS
ISOPAR M	218–257	96	97.65	Emulsion	0.09	CPS
ShellSol D70^®^	190–250	78	97.31	Emulsion	0.14	CPS
*n*-Dodecane	216	71	97.07	Emulsion	0.56	CPS
Escaid^TM^ 110	200–250	82	97.45	Emulsion	0.19	CPS
Kerosene +20% *n*-Octanol	—	—	99.81	CPS	2.22	CPS
ISOPAR M +20% *n*-Octanol	—	—	99.75	CPS	1.91	CPS
ShellSol D70^®^ + 20% *n*-Octanol	—	—	99.80	CPS	1.28	CPS
*n*-Dodecane + 20% *n*-Octanol	—	—	99.73	CPS	0.34	CPS
Escaid^TM^ 110 + 20% *n*-Octanol	—	—	99.74	CPS	0.67	CPS
Exxal^TM^ 10	216–226	90	99.78	CPS	1.35	CPS
*n*-Octanol	195	27.2	99.67	CPS	2.32	CPS
**Aromatic/chlorinated Diluents**						
*p*-Xylene	138	25	96.71	CPS	0.37	CPS
*o*-Dichlorobenzene	183.3	66	99.57	CPS	0.34	CPS
Toluene	110	4.4	96.66	CPS	0.45	CPS
CHCl_3_	61.0	—	99.85	CPS	0.33	CPS

CPS: Clear Phase Separation. Conditions: [Pd(II)] = 1 mM in 0.1 M HCl; [E] = 1 mM; Time = 30 min; O/A = 1; Shaking speed = 300 rpm.

Under these conditions, the extraction of Pd(II) is negligible, i.e., < 0.5%. Thus, extractant **1** shows very high Pd(II) extraction ability, whereas that of **2** is negligible. From these results, it is clear that **1** is effective for Pd(II) extraction in all the diluents investigated and is a suitable Pd(II) extractant for PGM recovery by liquid-liquid extraction.

### Effect of shaking time on the extraction of Pd(II)

**Figure 1:**
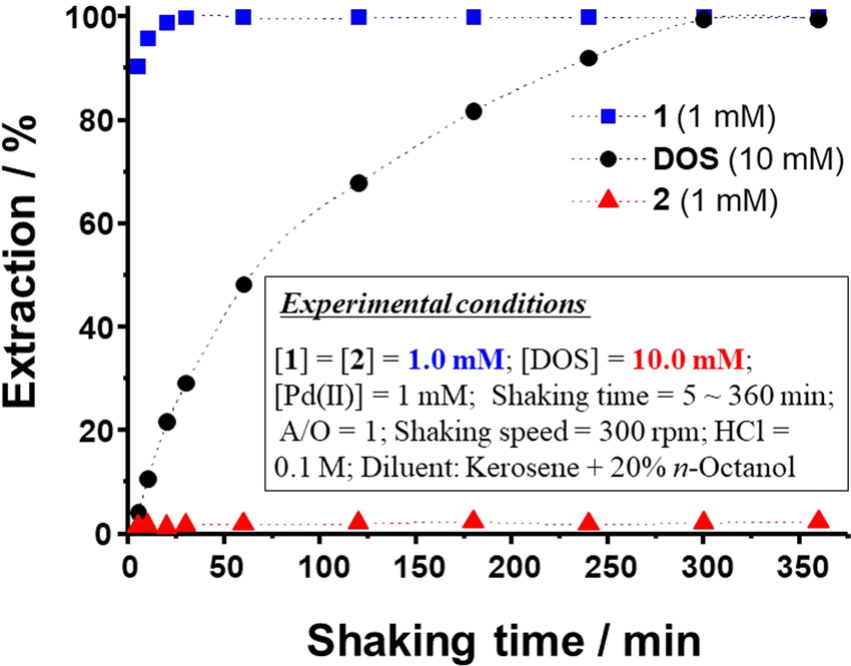
Effect of shaking time on Pd(II) extraction by **1** and **2**.

In industrial liquid-liquid extraction processes, extraction kinetics play a very important role. In order to compare the Pd(II) extraction kinetics of **1** and **2** with those of the commercial extractant DOS, the effect of shaking time from 5 min to 6 h was studied. In these studies, the concentration of DOS used was 10-fold those of **1** and **2** (1 mM) in order to unambiguously demonstrate the advantages of the new extractants. The effects of shaking time on the extraction of Pd(II) by **1**, **2**, and DOS are given in Fig. 1. The results indicate that **1** reaches saturation at 30 min with *E*% = 99.9%, whereas DOS attains saturation at 360 min with *E*% = 99.1%, demonstrating the comparatively poor extraction kinetics of DOS^11^. Thus, extractant **1** shows Pd(II) extraction kinetics 12-fold faster than those of DOS. Conversely, **2** exhibits only 2.2% Pd(II) extraction at 360 min. Hence, all further studies were conducted with a shaking time of 30 min. The higher extraction ability and rate of **1** are attributed to the efficient and rapid Pd(II) coordination by the sulfur atoms present in its thioamide groups.

### Extraction of Pd(II) as a function of HCl or HNO_3_ concentration

**Figure 2:**
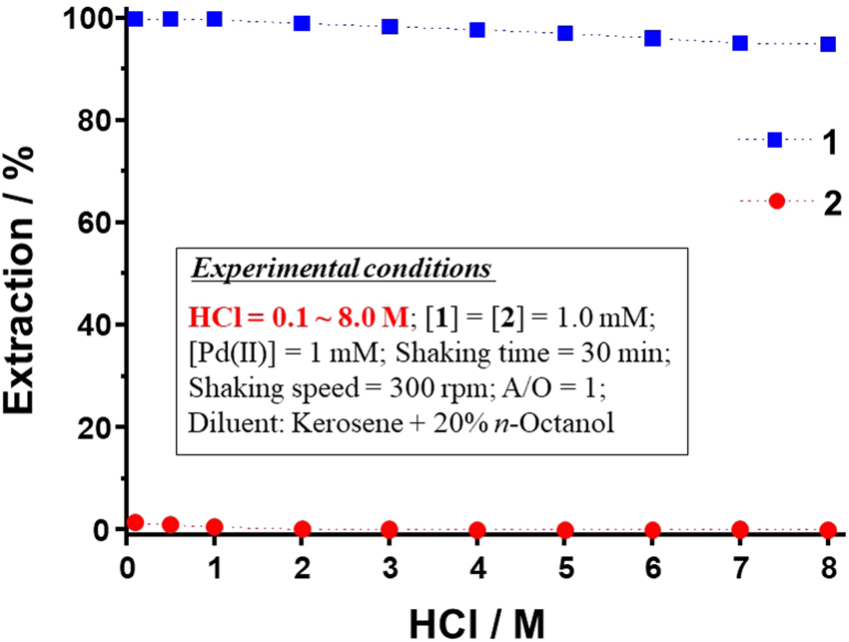
Effect of HCl concentration on Pd(II) extraction by **1** and **2**.

**Figure 3:**
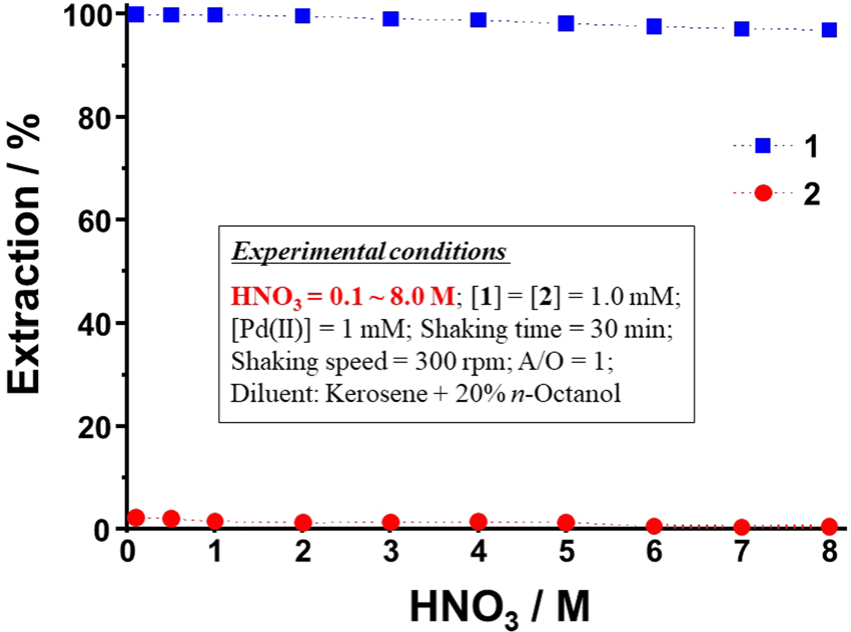
Effect of HNO_3_ concentration on Pd(II) extraction by **1** and **2**.

In hydrometallurgical extraction processes, metal ions are usually leached using very highly concentrated solutions of HCl or HNO_3_. Thus, the leach liquors are very acidic in nature (0.1‒8.0 M). Consequently, the newly synthesized extractants must be capable of extracting metal ions from acidic leach liquors. Therefore, Pd(II) extraction by **1** and **2** was conducted from 0.1‒8.0 M HCl or HNO_3_. The effects of HCl and HNO_3_ concentration, respectively, on Pd(II) extraction using **1** and **2** are illustrated in Figs 2 and 3. The Pd(II) extraction efficiencies for **1** range from 99.9‒94.8% for 0.1‒8.0 M HCl. In the case of HNO_3_, the Pd(II) extraction efficiencies for **1** range from 99.9‒96.9% for 0.1‒8.0 M HNO_3_. In both cases, Pd(II) extraction by **1** is not significantly affected by HCl or HNO_3_ concentration. Pd(II) is a soft acid and has a tendency to bind strongly to the soft-base sulfur donor atoms of **1**. The Pd(II) *E*% of **2** was found to be negligible (< 2%) from 0.1‒8.0 M HCl and HNO_3_. The amide-functionalized extractant **2** shows very poor Pd(II) extraction in the high-concentration HCl and HNO_3_ media, which may be due to the hard and soft acid and base (HSAB) properties of the amide functional groups of **2**. Specifically, in the case of amide groups, the donor oxygen atom acts as a hard base and, according to HSAB theory, will not bind well to the softly acidic Pd(II). Similar results have been reported previously for Pd(II) extraction^31–33^. Hence, all further Pd(II) extraction experiments were conducted with extractant **1** only.

### Acid stability of extractant 1

As revealed above, extractant **1** shows very efficient Pd(II) extraction in both HCl and HNO_3_ media. However, in order to apply the synthesized extractant **1** to industrial refining processes that typically involve long-run and repeated extractions, it must be very stable and durable in acid media. Therefore, the stability of extractant **1** was assessed in 12 M HCl and 1 M HNO_3_ over one week. The stability and deterioration of the acid-treated **1** was assessed using FT-IR. The FT-IR spectra of native **1** and acid-treated **1** are shown in Fig. S2. The FT-IR spectra of 12 M HCl- and 1 M HNO_3_-treated extractant **1** exactly match that of native **1**, indicating that **1** is very stable and that no deterioration occurs during acid treatment. Thus, because of extractant **1** is very high acid stability, it can be conveniently used for metal recovery from the highly acidic aqueous solutions produced in refineries.

### Optimal O/A phase ratio for effective Pd(II) extraction

**Figure 4:**
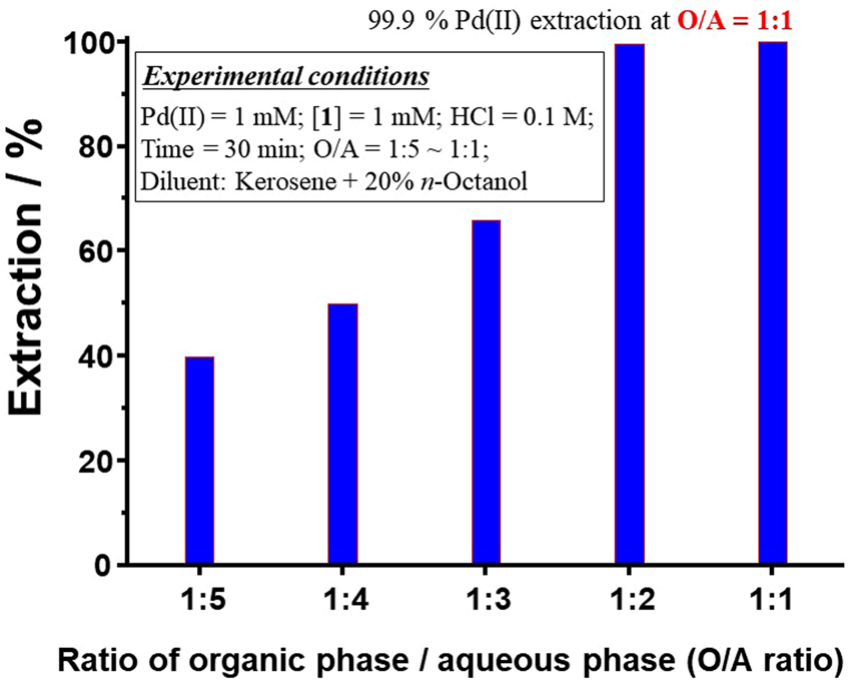
Effect of O/A ratio on Pd(II) extraction.

The use of the optimal O/A phase ratio is very important for effective Pd(II) extraction. Furthermore, the economy of the extraction process depends significantly on the O/A phase ratio. In the current study, the O/A phase ratio was varied from 1:5 to 1:1, and the results are shown in Fig. 4. At an O/A phase ratio of 1:5, Pd(II) extraction is 39.8%. O/A phase ratios of 1:4 and 1:3 result in Pd(II) *E*% values of 49.8% and 65.7%, respectively. For O/A phase ratios of 1:2 and 1:1, Pd(II) *E*% is 99.5% and 99.9%, respectively. These results clearly demonstrate that O/A phase ratios of 1:2 or 1:1 are sufficient for Pd(II) extraction efficiencies of > 99%. Conversely, for DOS, an O/A phase ratio of ≥ 10 is required for complete Pd(II) extraction (*cf*. Fig. 1). The current results indicate that an O/A phase ratio of 1 is optimal for complete Pd(II) extraction from aqueous solutions.

### Determination of Pd(II) distribution ratio (*D*)

**Figure 5:**
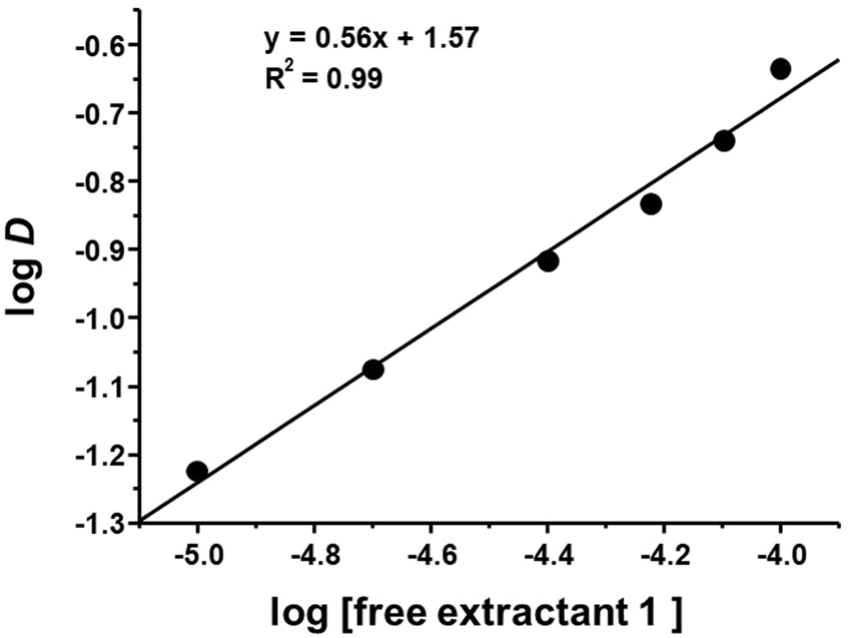
Plot of log *D* vs. log [free extractant **1**] for Pd(II) extraction. Conditions: [**1**] = 0.01–0.1 mM; Pd(II) = 1.0 mM in 0.1 M HCl; O/A = 1; shaking time = 30 min; shaking speed = 300 rpm; diluent = kerosene + 20% *n*-octanol; temperature = 20 ± 1 °C.

The Pd(II) distribution ratio was confirmed by determining the distribution ratio (*D*) between the extractant and Pd(II). The Pd(II) distribution ratio was determined using 1 × 10^−5^–1 × 10^−4^ M **1** with 1.0 mM Pd(II) in 0.1 M HCl shaken for 30 min. A plot of log *D* vs. log [**1**] was drawn, and the slope was calculated. The log-log plot of Pd(II) distribution against the concentration of **1** is shown in Fig. 5. The plot presents a straight line with a slope value of 0.56, which clearly indicates that the stoichiometry of the extracted species is one mole of extractant **1** and two moles of Pd(II), i.e., a complex with a **1**:Pd(II) ratio of 1:2. Thus, the result Pd(II) distribution ratio experiment indicate that extractant **1** extracts Pd(II) via a 1:2 extractant-Pd complex. This demonstrates that extractant **1** is an economical extractant as compared to the commercial extractant DOS currently used for Pd(II) extraction.

### Extraction of Pd(II) by 1 from a simulated mixed PGM and base metal solution

**Figure 6:**
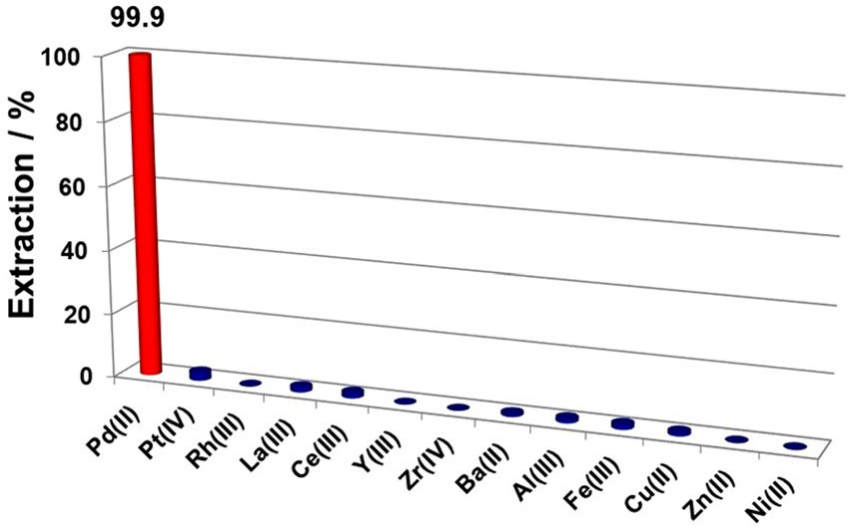
Extraction of Pd(II) by **1** from a simulated mixed-metal solution. Conditions: Metal ions = 100 mg/L each; [HCl] = 0.5 M; [**1**] = 1 mM; diluent = kerosene + 20% *n*-octanol; time = 30 min; O/A = 1; shaking speed = 300 rpm.

In order to study the selectivity for Pd(II) of **1**, a liquid-liquid extraction was conducted from a simulated mixed-metal solution. Specifically, a 10 mL sample of a simulated mixed-metal solution containing 100 mg/L each of Pd, Pt, Rh, Y, Zr, Ba, Al, La, Ce, Fe, Ni, Cu, and Zn in 0.5 M HCl was shaken with 10 mL of 1 mM **1** in kerosene containing 20% *n*-octanol for 30 min. The results for the extraction of Pd(II) from the simulated solution by **1** are shown Fig. 6. Extractant **1** selectively extracts only Pd(II) over other metals with a Pd(II) *E*% > 99.9%. The *E*% values for all the other metals were found to be < 1%. Thus, extractant **1** shows a clear selectivity toward Pd(II) ions over Pt, Rh, Y, Zr, Ba, Al, La, and Ce, and the base metals Fe, Ni, Cu and Zn. The results of the study clearly demonstrate that extractant **1** is highly suitable for the separation of Pd(II) from other PGMs and base metals.

### Extraction of Pd(II) by 1 from an automotive catalyst leach liquor and its reusability

**Figure 7:**
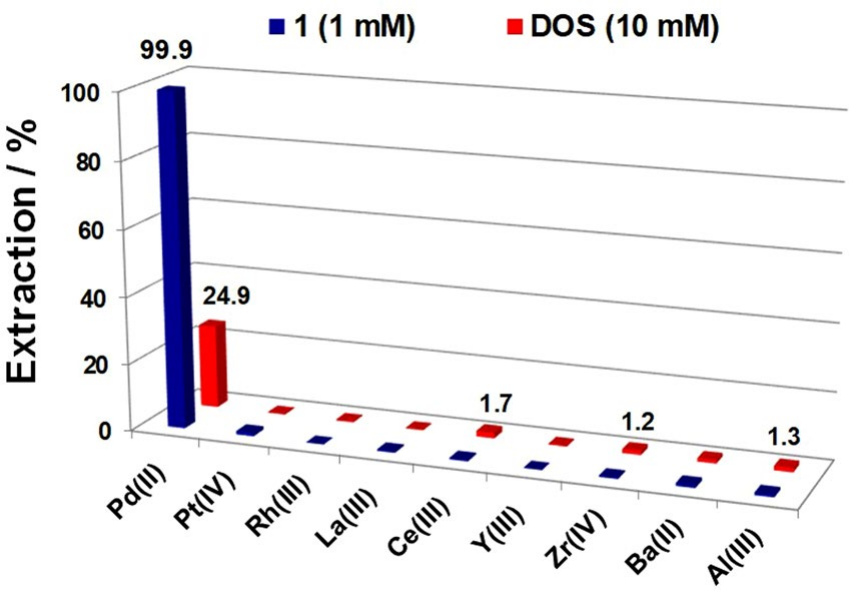
Metal ion *E*% values for **1** and DOS from a catalyst leach liquor. Conditions: shaking time = 30 min; [**1**] = 1.0 mM; [DOS] = 10 mM; pH = 1.22 (~0.06 M HCl); O/A = 1; shaking speed = 300 rpm.

An automotive catalyst residue, a secondary resource for PGMs, was procured from a commercial source in Japan. Automotive catalysts typically contain 1‒2% PGMs and 90% supporting materials such as La_2_O_3_, CeO_2_, ZrO_2_, Al_2_O_3_, BaO, and other metal oxides. First, the automotive catalyst residue was pre-treated with hydrogen reduction and then milled. The leaching of the milled material was performed using HCl (11.6 M) + H_2_O_2_ (1 vol%), and the resultant leachate was then characterized using an inductively coupled plasma atomic emission spectrometer (ICP-AES). The automotive catalyst leachate contained Pd, Pt, Rh, Y, Zr, Ba, Al, La, and Ce (Table S1). The extraction of Pd(II) from the leach liquor was attempted using 1 mM **1** or 10 mM DOS in kerosene containing 20% *n*-octanol or Exxal^TM^ 10 alone as diluents with the leach liquors diluted five times with water. Liquid-liquid extractions from the leach liquor using **1** or DOS were conducted for 30 min at 300 rpm. The metal *E*% values obtained using **1** and DOS are given in Fig. 7. Extractant **1** selectively extracts 99.9% of the Pd(II) from the leach liquors, whereas commercial DOS extracts only 25%. *E*% values for all the other metal ions present in the leach liquor were found to be < 2%. **1** diluted in Exxal^TM^ 10 shows a similar Pd(II) extraction performance, i.e., *E*% = 99.9% for Pd(II) and < 2.4% for the other metals ions. Pd(II) *E*% for **1** in Exxal^TM^ 10 is shown in Fig. S3.

Furthermore, stripping of Pd(II) from the kerosene/*n*-octanol phase after leaching was performed using 1 M HCl, 1 M HNO_3_, 10% (*v/v*) NH_3_, or 0.1 M thiourea + 1.0 M HCl. The Pd(II) stripping efficiencies (*S*%) using 1 M HCl, 1 M HNO_3_, or 10% (*v/v*) NH_3_ were found to be only 15‒20%, as shown in Table S2. However, in the case of the 0.1 M thiourea + 1.0 M HCl solution, *S*% was found to be 99.9%. Fig. S4(a) shows that this thiourea/HCl solution completely strips Pd(II) to the solution, whereas 10% (*v/v*) NH_3_ solution is not an effective stripping agent for Pd(II) (Fig. S4(b)). Several other researchers have also reported that a mixture of thiourea and HCl is an efficient stripping agent for Pd(II)^9,14,25–28^.

**Figure 8:**
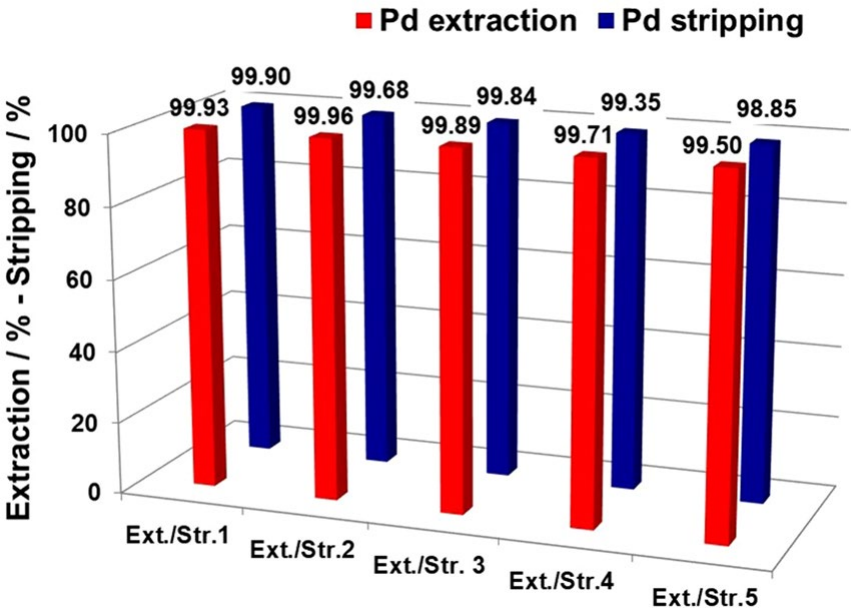
Extraction-stripping cycles from automotive catalyst leach liquor for extractant **1**. Conditions: Acid-leached liquor diluted 5-times with water; [HCl] = 0.06 M; [**1**] = 1 mM; diluent = kerosene + 20% *n*-octanol; time = 30 min; O/A = 1; shaking speed = 300 rpm; stripping aqueous phase = 0.1 M thiourea in 1.0 M HCl.

After the organic phase was washed with water to remove the thiourea, it was reused for the next cycle. In the present study, up to five Pd(II) extraction-stripping cycles were conducted. The results for the extraction-stripping cycles using **1** are exhibited in Fig. 8. The high *E*% (> 99%) and *S*% (> 98.9%) for Pd(II) exhibited by **1** allow its facile reuse for more than five cycles. Thus, extractant **1** shows very selective Pd(II) extraction and it may be reused many times in industrial applications.

### Mechanism of Pd(II) extraction by 1

**Figure 9:**
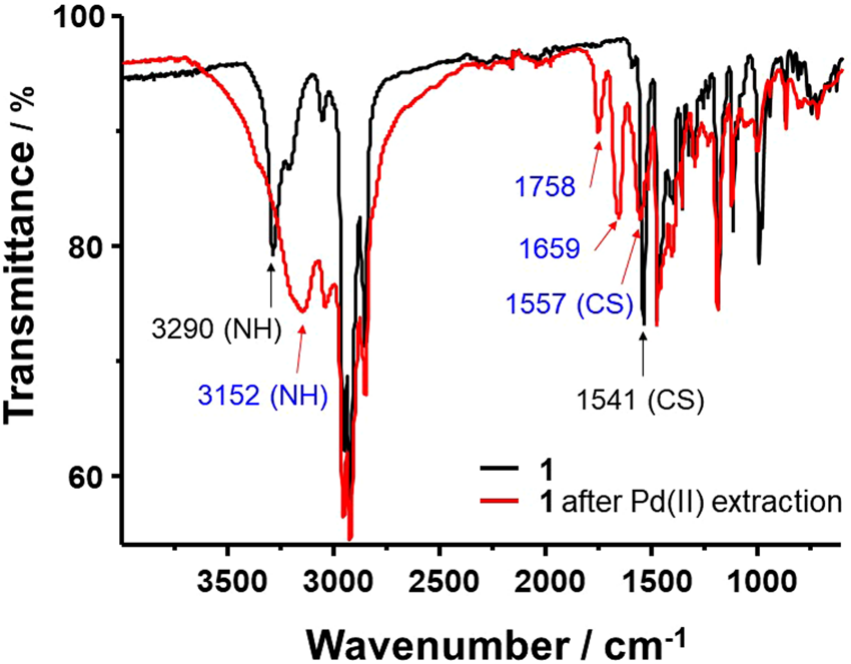
FT-IR spectra of **1** and **1**-Pd(II) complex.

A suitable **1**-Pd(II) complex for spectroscopic analysis was prepared using 1 mM **1** in CHCl_3_ with 1 mM Pd(II) in 0.1 M HCl. After Pd(II) extraction, the CHCl_3_ layer was evaporated to dryness to give the **1**-Pd(II) complex, and then FT-IR spectra were recorded. Fig. 9 shows the FT-IR spectra of **1** and the **1**-Pd(II) complex. The FT-IR spectrum of extractant **1** alone is significantly different from that after coordination with Pd(II), and several new peaks appear. The peak corresponding to -N‒H stretching is shifted from 3290 to 3152 cm^−1^ and the peak corresponding to -C=S is shifted from 1541 to 1557 cm^−1^. There is no free ligand in the **1**-Pd(II) complex and the peak assigned to C=S in the free ligand completely disappeared in the Pd(II) complexation.

**Figure 10:**
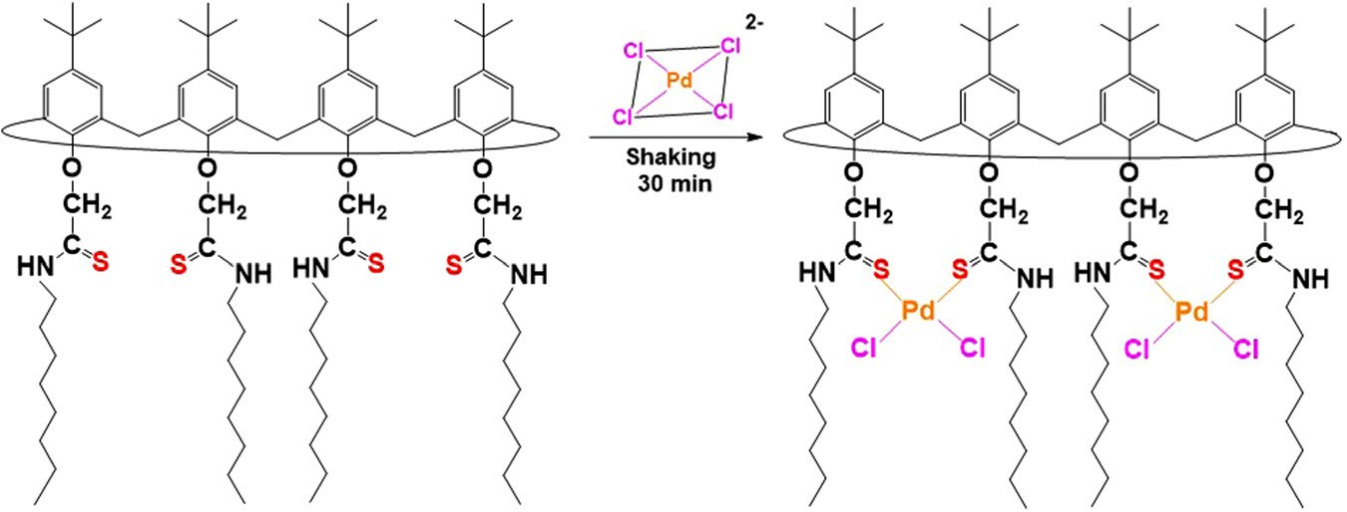
Proposed Pd(II) extraction mechanism for extractant **1**.

Thus, the results of these FT-IR studies show that the calixarene thioamide moieties of **1** capture Pd(II) ions via coordination^26,27^. Based on all of the results, including those of log-log plot and FT-IR analysis, a rational Pd(II) extraction mechanism for extractant **1** is proposed as illustrated in Fig. 10.

## Methods

### Materials and methods

*p*-*tert*-Butylphenol, HCHO, NaOH, NaH, ethyl bromoacetate, ammonium chloride, CHCl_3_, and diethyl ether were purchased from Kanto Chemical Co., Inc., Japan. Lawesson’s reagent, *n*-octanol, *n*-octylamine, and DOS were purchased from Tokyo Chemical Industry Co., Ltd. PGM and other metal solutions were prepared using PtCl_4_ (Acros Organics), PdCl_2_, NiCl_2_·6H_2_O, ZnCl_2_ (Kanto Chemical Co., Inc.), RhCl_3_·3H_2_O, FeCl_3_, CuCl_2_·2H_2_O, AlCl_4_, YCl_3_·6H_2_O, ZrCl_4_, LaCl_3_·7H_2_O, BaCl_2_·2H_2_O (Wako Pure Chemical Industries, Ltd.), and CeCl_3_·7H_2_O (Nacalai Tesque, Inc.) in acidic solution. The commercial hydrocarbon fluids, Exxal^TM^ 10 (isodecyl alcohol), and Escaid^TM^ 110 fluid (light hydrocarbons) were obtained from ExxonMobile Chemical Asia Pacific, Singapore. The other commercial diluents, i.e., kerosene (Nacalai Tesque, Inc., Japan), ShellSol D70 (Kremer Pigmente, GmbH & Co. KG), and ISOPAR M (Tonen General Petroleum Co., Ltd., Tokyo) were used as received. All other chemicals were obtained from commercial sources. The concentrations of the metal ions were obtained ICP-AES (SPS-3000, Seiko Instruments Inc. Japan). FT-IR spectra of the extractants and their metal ion complexes were obtained using a Nicolet iS5 spectrophotometer (Thermo Fisher Scientific, Tokyo). ^1^H NMR and ^13^C NMR data were obtained using a DPX 300 (Bruker, USA). Elemental analysis (C, H, N) of the extractants was performed using a CE-440 M elemental analyzer.

### Synthesis of extractants 1 and 2

**Figure 11:**
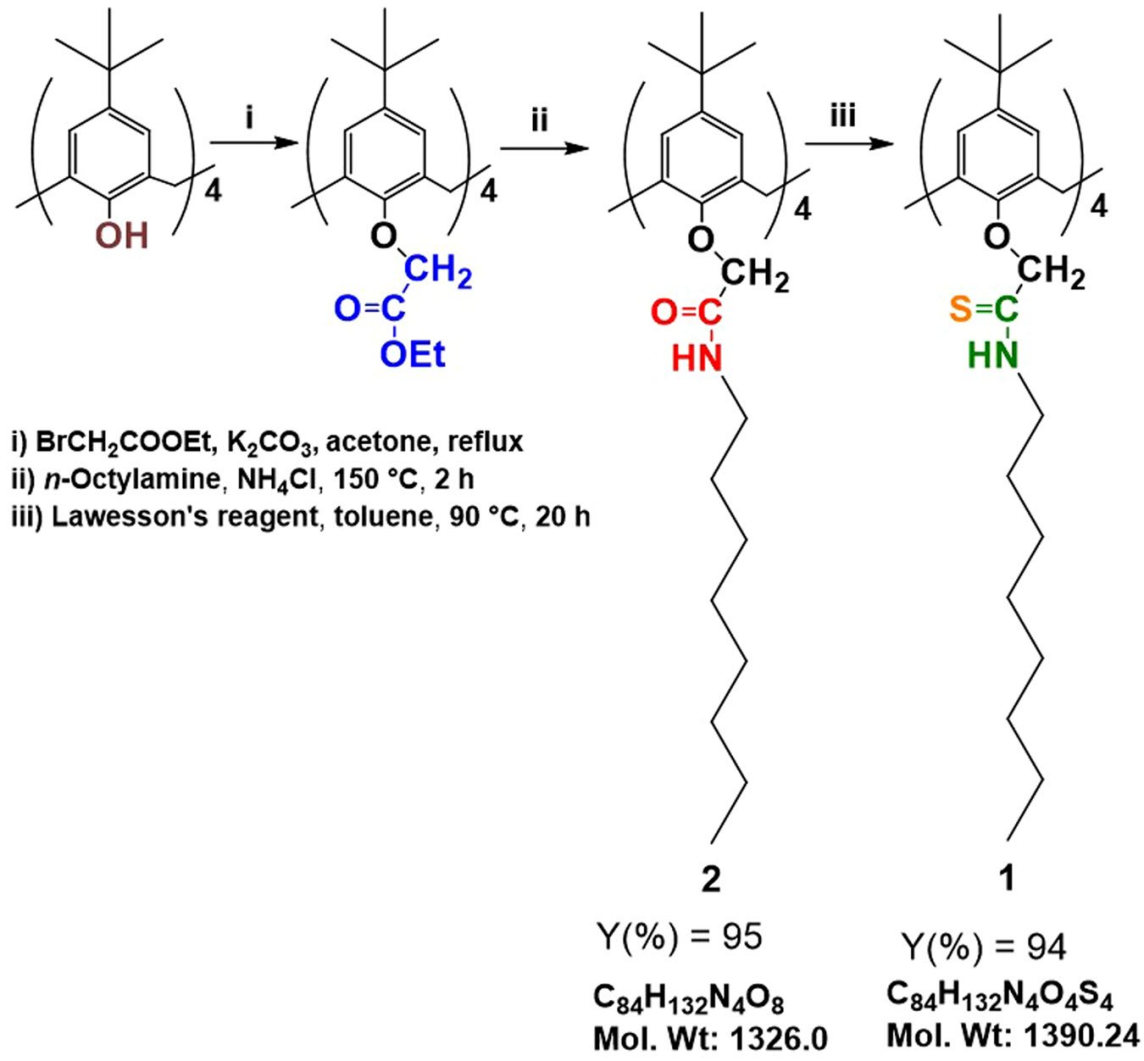
Synthesis of amide-modified calix[4]arene (**2**) and thioamide-modified calix[4]arene (**1**).

*p*-*tert*-Butylcalix[4]arene and 4-*tert*-butylcalix[4]arene-tetraacetic-acid tetraethyl ester were synthesized in good yields according to literature procedures^16,34^. The synthesis of extractants **1** and **2** is illustrated in Fig. 11. 25,26,27,28-Tetrakis[(*N*-*n*-octylcarbamoyl)methoxy-5,11,17,23-tetra-*tert*-butylcalix[4]arene (**2**) was first synthesized by Cho *et al*.^35^ by the direct reaction of *p*-*tert*-butylcalix[4]arene and 2-bromo-*N*(*n*-octyl)acetamide. However, owing to the expense and poor commercial availability of 2-bromo-*N*(*n*-octyl)acetamide, compound **2** was synthesized by an alternative route from previous literature^36^ as follows: 4-*tert*-Butylcalix[4]arene-tetraacetic acid tetraethyl ester (0.109 g, 0.11 mmol) and ammonium chloride (1.28 mg, 0.024 mmol) were placed into a round-bottom flask, and *n*-octylamine (0.284 g, 2.2 mmol) was added. The mixture was stirred at 150 °C for 2 h. The reaction mixture was then cooled to room temperature and ethanol (30 mL) was added. The resultant residue was filtered and dried *in vacuo* at 100 °C. The target compound **2** was obtained as a white crystalline solid (0.138 g, 95%)^36^. ^1^H NMR (CDCl_3_; 300 MHz): δ 7.47 (br t, 4H; N*H*), 6.77 (s, 8H; Ar*H*), 4.49 (s, 8H; OC*H*_2_), 4.46 (d, 4H; ArC*H*_2_Ar), 3.38–3.31 (m, 8H; NC*H*_2_), 3.24 (d, 4H; ArC*H*_2_Ar) 1.60–1.57 (m, 8H, C*H*_2_), 1.35–1.23 (m, 40H; C*H*_2_), 1.07 (s, 36H; C*H*_3_) 0.88 (t, 12H; C*H*_3_). FT-IR: *ν* (cm^−1^) 3290 (N-H), 1652 (C=O).

25,26,27,28-Tetrakis[(*N*-*n*-octylthiocarbamoyl)methoxy-5,11,17,23-tetra-*tert*-butylcalix[4]arene (**1**) was synthesized as follows: To a solution of **2** (0.044 g, 0.033 mmol) in dry toluene (10 mL) was added 0.035 g (0.089 mmol) of Lawesson’s reagent. The mixture was heated for 20 h at 90 °C. The toluene was then removed *in vacuo*, and the residue was extracted with CH_2_Cl_2_. The combined CH_2_Cl_2_ extracts were washed with water, dried over MgSO_4_, and evaporated *in vacuo*. The crude product was triturated with MeOH to give 0.043 g (94.0%) of compound **1** as a white solid. ^1^H NMR (300 MHz, CDCl_3_, TMS): δ 8.62 (br t, 4H; N*H*), 6.79 (s, 8H; Ar*H*), 4.91 (s, 8H; OC*H*_2_), 4.36 (d, 4H; ArC*H*_2_Ar), 3.80–3.75 (m, 8H; NC*H*_2_), 3.35 (d, 4H; ArC*H*_2_Ar), 1.64–1.57 (m, 8H, C*H*_2_), 1.36–1.28 (m, 40H; C*H*_2_), 1.08 (s, 36H; C*H*_3_) 0.88 (t, 12H; C*H*_3_). ^13^C NMR (75 MHz, CDCl_3_, TMS): δ 197.0, 151.5, 146.2, 131.9, 126.2, 80.7, 45.8, 33.9, 32.2, 31.8, 31.2, 29.2, 27.9, 27.0, 22.06, 14.1. FT-IR: *ν* (cm^−1^) 3290.2 (NH), 1541.7 (C=S). Anal. Calcd for C_84_H_132_O_4_N_4_S_4_: C, 72.57; H, 9.57; N, 4.03. Found: C, 72.36; H, 9.59; N, 4.10. The structure of compound **1** was confirmed using single-crystal X-ray crystallography. A single crystal of **1** was grown in 1:1 CHCl_3_ and MeOH at room temperature. X-ray diffraction data for **1** was collected using a Rigaku Saturn 724 CCD diffractometer with MoKα radiation. The detailed X-ray crystallography measurement procedure is given in the Supporting Information. The partial crystal structure was derived as shown in Fig. S5. The crystal structure of **1** was found to resemble a pinched cone with disordered *n*-octyl groups.

### Liquid-liquid extraction procedure

The metal extraction experiments were performed using pre-determined concentrations of the extractants in various diluents and PGM solutions in HCl/HNO_3_ or leach liquors of automotive catalysts of the desired concentrations (mM). For all the extraction studies, O/A phase ratio of 1 (i.e., 1:1, *v/v*) was maintained (except for the experiments on the O/A phase ratio) and the standard deviations of the extraction percentages were within ± 5%. All the extraction experiments were conducted at 20 ± 1 °C. The effect of diluent on Pd(II) extraction by **1** and **2** was investigated using 1 mM Pd(II) in 0.1 M HCl and 1 mM solutions of the respective extractants shaken for 30 min. The effect of shaking time on the extraction of Pd(II) was investigated using 1 mM Pd(II) in 0.1 M HCl with 1 mM **1** or **2** (or 10 mM DOS for comparison purposes) diluted in kerosene containing 20% *n*-octanol shaken for 5–360 min. The effect of HCl and HNO_3_ concentration on Pd(II) extraction was performed using 0.1–8.0 M HCl or HNO_3_ using 1 mM **1** and **2** in kerosene containing 20% *n*-octanol for 30 min. The distribution ratio (*D*) of Pd(II) with **1** was calculated using slope analysis^18,19^. The slope analysis was performed using 0.01–0.1 mM **1** with 1.0 mM Pd(II) in 0.1 M HCl. The appropriate O/A phase ratio for the Pd(II) extraction was determined by varying the O/A phase ratio from 1:5 to 1:1 using 1 mM **1** with 1 mM Pd(II) in 1.0 M HCl. Pd(II) extraction from a simulated mixed-metal solution containing of 100 mg/L each of Pt, Rh, Y, Zr, Ba, Al, La, and Ce and the base metals Fe, Cu, Zn, and Ni in 0.5 M HCl using 1 mM **1** for 30 min (the 12 metals above are generally used for the preparation of simulated automotive catalyst converter residue solutions) was also investigated. The acid stability of **1** was assessed over one week using 12 M HCl and 1 M HNO_3_ using 10 mM **1** diluted in CHCl_3_ with an O/A ratio of 1.

The extraction percentages (*E*%) were determined using equations 1 and 2:

1 $$E \% ={[{\rm{M}}]}_{{\rm{org}}}/{[{\rm{M}}]}_{{\rm{aq}},{\rm{init}}}\times 100$$

2 $${[{\rm{M}}]}_{{\rm{org}}}=({[{\rm{M}}]}_{{\rm{aq}},{\rm{init}}}-{[{\rm{M}}]}_{{\rm{aq}}})$$

where [M]_aq,init_ and [M]_aq_ are the initial and final concentrations of the metal ions in the aqueous solutions, respectively. During extraction, the volumes of the organic and aqueous phases did not change. The distribution ratio (*D*) of Pd(II) between the organic and aqueous phases was calculated using equation 3.

3 $$D={[{\rm{M}}]}_{{\rm{org}}}/{[{\rm{M}}]}_{{\rm{aq}}}$$

### Pd(II) extraction from automotive catalyst leach liquors

Automotive catalyst residues were leached with HCl (11.6 M) + H_2_O_2_ (1 vol%) as per our previously reported procedure^37^. The acid-leached automotive catalyst residue liquids were diluted five times with distilled water, and the metal ion concentrations were measured using ICP-AES. The concentrations of the metal ions in the leach liquids (mg/L) were Pd(II) = 92.8, Pt(IV) = 54.2, Rh(III) = 37.2, La(III) = 86.7, Ce(III) = 608.8, Y(III) = 3.8, Zr(IV) = 25.6, Ba(II) = 289.3, and Al(III) = 320.9. The pH of the leach liquors was 1.22 (~0.06 M HCl). Pd(II) extraction studies were performed using 10 mL of diluted automotive catalyst leach liquid with 10 mL of 1 mM **1** (or 10 mM DOS) in either kerosene containing 20% *n*-octanol or unadulterated Exxal^TM^ 110 shaken for 30 min.

### Recovery of Pd(II) and reusability of extractant 1

The Pd(II) extraction reusability of **1** was assessed using leach liquors over five extraction-stripping cycles. Pd(II) extraction from the leach liquors was carried out according to the method outlined in previous section. Then, the Pd(II) was stripped from 10 mL of the resulting extractant media (organic phase) with 10 mL of 1 M of HCl, 1 M HNO_3_, 0.1 M thiourea in 1.0 M HCl, or 10% (*v/v*) aqueous NH_3_. After stripping the Pd(II) from the organic phase, the organic phase was washed with 20 mL of water in order to remove the stripping agent. The water washed extractant phase was used for further extraction-stripping cycles. The stripping ability, *S*%, was calculated using Equation 4:

4 $$S{\rm{ \% }}={[{\rm{P}}{\rm{d}}({\rm{I}}{\rm{I}})]}_{{\rm{a}}{\rm{q}}}/{[{\rm{P}}{\rm{d}}({\rm{I}}{\rm{I}})]}_{{\rm{o}}{\rm{r}}{\rm{g}}}\times 100$$

where [Pd(II)]_aq_ is the concentration of the Pd(II) ions in the aqueous solution after back-extraction, and [Pd(II)]_org_ is the concentration of Pd(II) in the organic phase before back-extraction. The volumes of the organic and aqueous phases did not change during extraction.

## Conclusions

A novel long-alkyl-chain-thioamide-functionalized calix[4]arene (**1**) was synthesized for selective Pd(II) separation from automotive catalyst residue leachates. Extractant **1** in various aliphatic and aromatic/chlorinated diluents exhibits very high Pd(II) extraction ability (> 99%) in only 30 min shaking time. The Pd(II) extraction kinetics of **1** were found to be 12-fold faster than those of DOS. Extractant **1** shows 99.9‒94.8% Pd(II) extraction in both 0.1‒8.0 M HCl and 0.1‒8.0 M HNO_3_ media, and was found to be very stable in those media. The Pd(II) distribution ratio of **1** was found to be 1:2 (extractant-Pd complex). Extractant **1** in kerosene containing 20% *n*-octanol exhibits selective extraction of Pd(II) (*E*% = 99.9%) from simulated mixed-metal solutions and automotive catalyst acid-leached liquors. Effective extraction (*E*% = 99.9‒99.5%) and stripping of Pd(II) (*S*% = 99.9‒98.8%) from the leachates were achieved for five extraction-stripping cycles, and after the organic phase was stripped of Pd(II), it could be reused for Pd(II) extraction. FT-IR studies revealed that **1** extracts Pd(II) via coordination through its thioamide functional groups. Thus, extractant **1** is proposed as a new macrocyclic extracting reagent for the separation and recovery of Pd(II) from primary and secondary resources in hydrometallurgy-based PGM refineries.

## Electronic supplementary material

Supplementary Information File
