# Supplementary material for: Rapid and selective recovery of palladium from platinum group metals and base metals using a thioamide-modified calix[4]arene extractant in environmentally friendly hydrocarbon fluids
Source: Sci Rep. 2018 Nov 15;8:16909. doi: 10.1038/s41598-018-35026-x (PMC6238000; doi:10.1038/s41598-018-35026-x)
Supplement: Supplementary file 1 — Supplementary Information File [file 41598_2018_35026_MOESM1_ESM.docx]

**Supporting Information**

**Rapid and selective recovery of palladium from platinum group metals and base metals using a thioamide-modified calix[4]arene extractant in environmentally friendly hydrocarbon fluids**

**Manabu Yamada ^1†^**, **Muniyappan Rajiv Gandhi ^2^**^†§^ **& Atsushi Shibayama ^2^**

^1^ Research Center of Advanced Materials for Breakthrough Technology, Graduate School of Engineering Science, Akita University, Akita 010-8502, Japan.

^2^ Graduate School of International Resource Sciences, Akita University, Akita 010-8502, Japan.

^†^These authors contributed equally to this work. ^§^Current address: Quality Control Department, Panipat Refinery & Petrochemical Complex, Indian Oil Corporation Limited, Haryana 132140, India. Correspondence and requests for materials should be addressed to M.Y. (email: myamada@gipc.akita-u.ac.jp)

**Contents**

Preliminary data of X-ray crystallography measurement of crystals 1

**Supporting Tables**

**Table S1.** The concentrations of metal ions in the leached liquors of automotive catalysts after 5 times dilution.

**Table S2.** Stripping of Pd(II) from the automotive leached solution extracted in kerosene + 20% *n*-octanol phase of **1** by 1 M HCl, 1 M HNO_3_, 10% (*v/v*) NH_3_ aqueous solution and 0.5 M thiourea + 1.0 M HCl solution.

**Supporting figures**

**Figure S1.** Extraction of Pd(II) by **1** in kerosene, kerosene + 20% *n*-octanol and extraction

of Pd(II) by **2** in kerosene.

**Figure S2.** The FT-IR spectra of native **1** and acid treated **1**.

**Figure S3.** Metal ions *E%* of **1** in Exxal^TM^ 10 from catalyst leach liquors.

Conditions: shaking time = 30 min; [**1**] = 1.0 mM in Exxal^TM^ 10; pH = 1.22

(~0.06 M HCl); O/A = 1; shaking speed = 300 rpm.

**Figure S4.** (a) Stripping of Pd(II) using 0.1 M thiourea/HCl (TU), (b) stripping of Pd(II)

using 10% (v/v) NH_3_.

**Figure S5.** Stick diagram of **1**. S = yellow, O = red, N = pale purple, C = dark grey (The H

atoms have been omitted for clarity).

**Preliminary data of X-ray crystallography measurement of crystals 1**

Single crystals of **1** for X-ray diffraction studies were grown from CHCl_3_ by slow evaporation at room temperature. The crystals in mother liquid were picked up with a pipette, and dropped in Paratone-N oil. The single crystals coated with oil were isolated on MicroMounts^TM^, and the crystals were placed in a cold nitrogen steam at 93 K. X-ray diffraction data were collected a Rigaku Saturn 724 CCD diffractometer with Mo-K*α* radiation (*λ* = 0.71073 Å). The structures were solved by direct methods using SHELXT-2014,^1^ and refined using the full-matrix least-squares method on *F*^2^ using SHELXL-2014.^2^ All materials for publication were prepared by Yadokari-XG 2009 software.^3,4^

**Crystal data.** C_85.5_H_132.5_Cl_1.50_N_4_O_5_S_4_, *M* =1477.95, colorless, crystal dimensions 0.35 × 0.35 × 0.10 mm^3^, triclinic, space group *P -*1, crystal description block, *a* = 13.4706(4), *b* = 18.5354(5), *c* = 19.9039(6) Å, *α* = 112.967(3)°, *β* = 91.894(2)^o^, *γ* = 97.076(2)°, *V* = 4522.9(2) Å^3^, *Z* = 1, Mo Kα radiation (λ = 0.71075 Å), *ρ*_calcd_ = 1.082 g/cm^3^, *T* = 93 K, *µ*(Mo Kα) = 0.197 mm^-1^, 60181 measured reflections, 20511 unique reflections (*R_int_ =* 0.0373), 15535 observed reflections (*I* > 2*σ*(*I*)), 966 parameters, *R* = 0.1402, *wR* = 0.4487, refined against |*F*|, GOF = 1.884.

**References**

(1) Sheldrick, G. SHLEXT ‒ Integrated space-group and crystal-structure determination. *Acta Crystallogr. Sect. A Found. Adv.* **2015**, *71*, 3-8.

(2) Sheldrick, G. Crystal structure refinement with SHELXL. *Acta Crystallogr. Sect. C Struct. Chem.* **2015**, *71*, 3-8.

(3) Wakita, K. *Yadokari-XG, software for crystal structure analyses*. **2001**.

(4) Kabuto, C.; Akine, S.; Nemoto, T.; Kwon, E. Release of software (Yadokari-XG 2009) for crystal structure analyses. *Nippon Kessho Gakkaishi* **2009**, *51*, 218-224.

**Supporting Table**

**Table S1.** The concentrations of metal ions in the leached liquors of automotive catalysts

after 5 times dilution.

| Metal ions | [M]_aq,init_ (mg/L) |
| --- | --- |
| Pd  Pt  Rh  La  Ce  Y  Zr  Ba  Al | 92.8  54.2  37.2  86.7  608.8  3.8  25.6  289.3  320.9 |

**Table S2.** Stripping of Pd(II) from the automotive leached solution extracted in kerosene + 20% *n*-octanol phase of **1** by 1 M HCl, 1 M HNO_3_, 10% (*v/v*) NH_3_ aqueous solution and 0.5 M thiourea + 1.0 M HCl solution.

| **Stripping reagent** | **Pd(II) *S*%** |
| --- | --- |
| 0.1 M thiourea + 1.0 M HCl solution | 99.9 |
| 10% (*v/v*) NH_3_ aqueous solution | 20.1 |
| 1 M HNO_3_ | 16.4 |
| 1 M HCl | 15.3 |

Shaking time = 30 min; shaking speeds = 300 rpm; O/A= 1; temperature *=* 20 ± 1 °C.


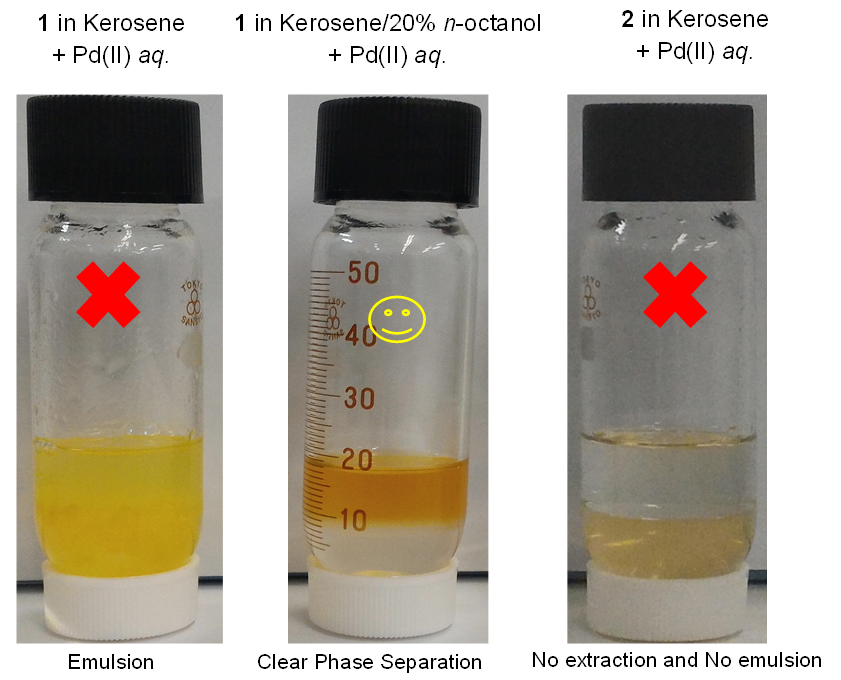


**Figure S1.** Extraction of Pd(II) by **1** in kerosene, kerosene + 20% *n*-octanol and extraction of Pd(II) by **2** in kerosene.

**Figure S2.** The FT-IR spectra of native **1** and acid treated **1**.

**Figure S3.** Metal ions *E%* of **1** in Exxal^TM^ 10 from catalyst leach liquors. Conditions: shaking time = 30 min; [**1**] = 1.0 mM in Exxal^TM^ 10; pH = 1.22 (~0.06 HCl); O/A = 1; shaking speed = 300 rpm.

**
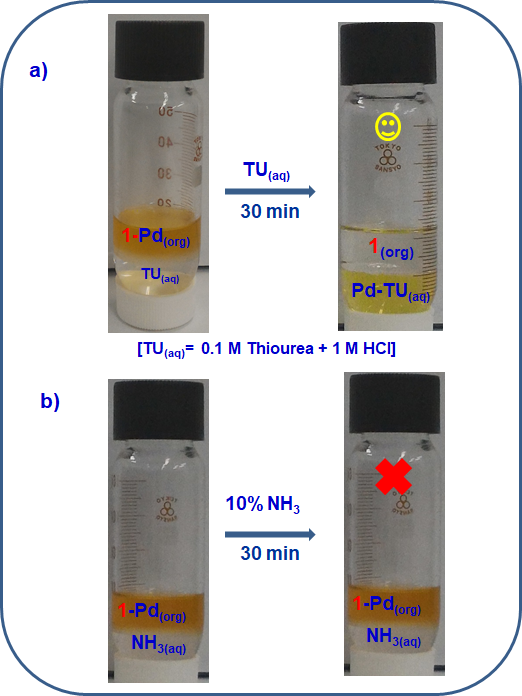
**

**Figure S4.** (a) Stripping of Pd(II) ion using 0.1 M thiourea/HCl (TU), (b) Stripping of Pd(II) ion using 10% (v/v) NH_3_.

**
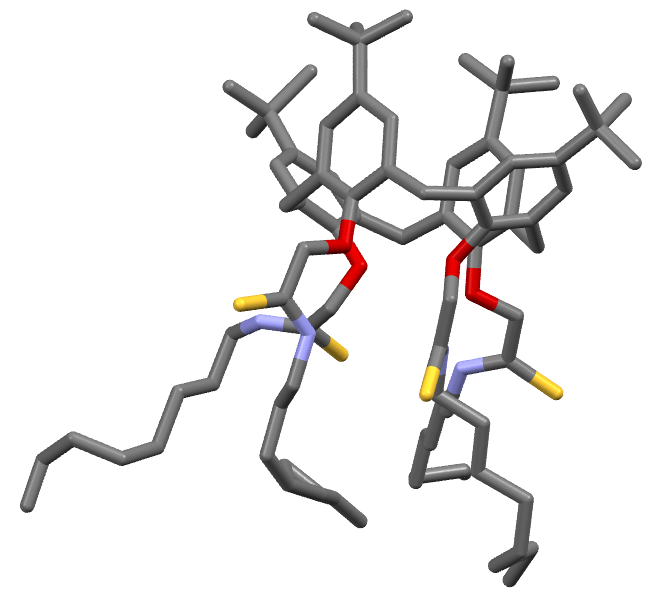
**

**Figure S5.** Stick diagram of **1**. S = yellow, O = red, N = pale purple, C = dark grey (The H atoms have been omitted for clarity).
